# Genomic inbreeding coefficients based on the distribution of the length of runs of homozygosity in a closed line of Iberian pigs

**DOI:** 10.1186/s12711-015-0153-1

**Published:** 2015-10-16

**Authors:** Luis Gomez-Raya, Carmen Rodríguez, Carmen Barragán, Luis Silió

**Affiliations:** Departamento de Mejora Genética Animal, Instituto Nacional de Investigación y Tecnología Agraria y Alimentaria (INIA), Ctra. de La Coruña km 7, 28040 Madrid, Spain

## Abstract

**Background:**

The increasing availability of DNA markers provides new metrics of inbreeding based on single nucleotide polymorphisms (SNPs), i.e. molecular inbreeding or the proportion of runs of homozygosity (ROH), as alternatives to traditional pedigree-based inbreeding coefficients. However, none of these metrics incorporate the length of ROH as an indicator of recent inbreeding. Novel inbreeding coefficients that incorporate length of ROH as a random variable with an associated density are investigated.

**Methods:**

New inbreeding metrics based on the distribution of the length of ROH are proposed: (1) the Kolmolgorov–Smirnov test, (2) a function of the quantiles of the cumulative distribution function of an individual versus the population, and (3) fitting of an exponential distribution to ROH lengths (mean, variance, and the probability of drawing at random a ROH larger than a given threshold). The new inbreeding and pedigree-based metrics were compared using 217 sows of an Iberian line that belong to three groups: C1 (conservation), C2 (conservation derived from C1), and S (selected and derived from C1), with complete pedigrees and genotyped for 35,023 SNPs.

**Results:**

Correlations between pedigree-based and the new genomic inbreeding coefficients ranged from 0.22 to 0.72 but most ranged from 0.60 to 0.70. The correlation between quantile chromosomal inbreeding coefficients (using molecular information of just one chromosome at the time) and chromosomal length was 0.84 (SE = 0.14), supporting the hypothesis that these coefficients incorporate information on ROH length as an indication of recent inbreeding. Kolmogorov–Smirnov and exponential chromosomal inbreeding coefficients were also correlated with chromosomal length (0.57). Chromosome 1 had the largest quantile ROH inbreeding coefficient (largest ROH sizes), whereas chromosome 10 had the lowest (shortest ROH sizes). Selection for lean growth increased ROH-based inbreeding coefficients for group S when compared to unselected groups C1 and C2. At the chromosomal level, this comparison showed that the level of autozygosity and the length of ROH for most of the autosomes increased in the selection line.

**Conclusions:**

Quantile and exponential probability inbreeding coefficients using ROH length as a random variable provide additional information about recent inbreeding compared to existing inbreeding coefficients such as molecular, pedigree-based or total ROH content inbreeding coefficients.

**Electronic supplementary material:**

The online version of this article (doi:10.1186/s12711-015-0153-1) contains supplementary material, which is available to authorized users.

## Background

The inbreeding coefficient of an individual is the probability that two alleles at a locus in that individual are identical by descent [1]. The inbreeding coefficient is a key parameter to understand the amount of matings between related individuals that have taken place in a population. Inbreeding leads to an increase in homozygosity, which, in turn, reduces performance of production traits (inbreeding depression), reduces fitness and compromises long-term viability of the population [2, 3]. Therefore, control of inbreeding is itself an objective in animal production or conservation genetics [4].

In farm animals, coefficients of inbreeding are systematically computed from pedigree records using path coefficients [5]. If pedigrees are not available, inbreeding coefficients can be calculated using molecular information. In particular, genome-wide single nucleotide polymorphism (SNP) bead chips are used to assess levels of homozygosity [6] or to estimate pedigree-based inbreeding coefficients [7, 8]. These approaches assume that SNPs are unlinked and they do not make use of all available information. However, SNPs are physically linked and alleles at linked markers on the same homologous chromosome are inherited together unless a recombination event occurs between them.

Runs of homozygosity (ROH) are defined as continuous and uninterrupted stretches of DNA sequences without heterozygosity in diploid state [9]. Presence of long ROH can imply recent inbreeding, which can be used to estimate genome-wide autozygosity and inbreeding coefficients, as suggested by Keller et al. [10]. ROH has been used to investigate inbreeding in human [11–13], cattle [14, 15], and pig populations [16, 17].

**Fig. 1:**
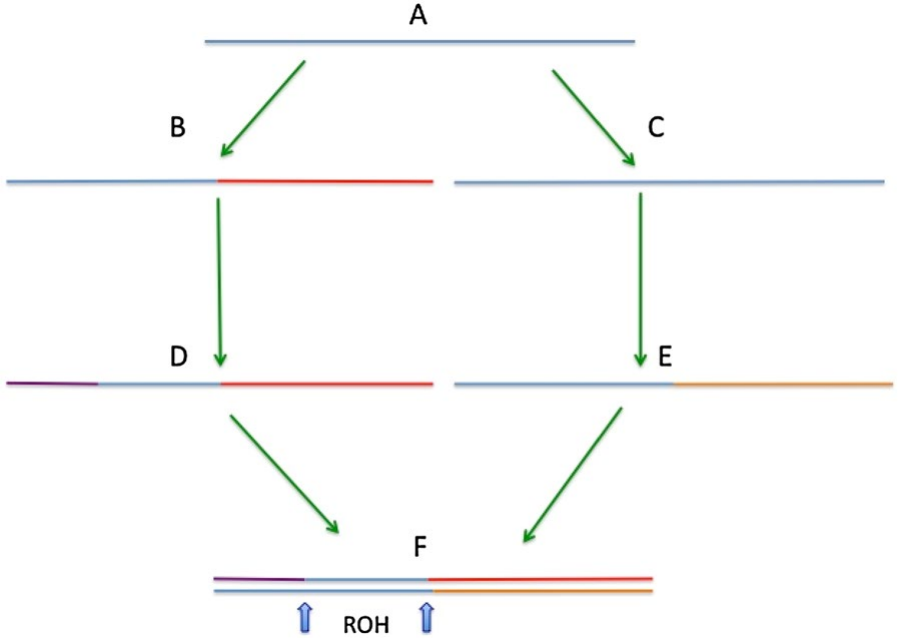
Illustration of the generation of a run of homozygosity. Individual *F* is the progeny from the mating between two related parents *D* and *E*, with a common ancestor *A*. Only one of the two homologous chromosomes (in *blue*) of ancestor *A* is represented

The generation of ROH is explained in Fig. 1. “A” represents a common ancestor of parents “D” and “E” of individual “F”. Individual “F” has a ROH fragment identical by descent, which is delimited by blue arrows. The line under “A” represents one of the two homologous chromosomes (in blue), which will generate the ROH in individual “F”. Colors other than blue are used to represent chromosomes of different origin. For this example, there were recombination events in paths “A” to “B”, “B” to “D”, and “C” to “E”; there were no recombination events in paths “A” to “C”, “D” to “F” or “E” to “F”. The effect of recombination is to break down the length of the homologous chromosome in steps from ancestor “A” to individual “F”. This illustration can be used to identify the main factors that affect the length of ROH: (a) the number of steps in the paths from “A” to “F” (opportunities for recombination, green arrows in the scheme), where a small number of steps (recent inbreeding) results in longer ROH; (b) the recombination rate in a chromosome (which can vary at the population or individual level); and (c) the length of the chromosome, with longer chromosomes yielding longer ROH because the longer the chromosome is in the ancestor, the longer is the ROH fragment in the individual in which inbreeding is assessed. However, the latter has not been proven empirically.

The approach used to compute inbreeding coefficients based on ROH requires calculating the total length of ROH covering the genome of an individual (for a given minimum number of contiguous homozygous SNPs) divided by the length of the genome [11, 18]. As stated above, recent inbreeding is associated to larger ROH fragments [10–19]. However, it is not well established either how to make a comparison between individuals with different numbers and lengths of ROH fragments or how to use the length of ROH to estimate recent inbreeding.

The objective of this paper was to investigate the use of ROH length as a random variable with an associated distribution or probability density to derive new inbreeding coefficients: (1) a method based on the Kolmogorov–Smirnov test, (2) a method based on quantiles of the distribution of the length of ROH, and (3) a method based on fitting an exponential distribution to the ROH-length distribution. These inbreeding coefficients were compared to SNP-based homozygosity metrics and pedigree inbreeding coefficients. It is shown that the new coefficients provide additional information on recent inbreeding. The new inbreeding coefficients were used to investigate inbreeding in a closed line of Iberian pigs maintained in a conservation program and to investigate the effect of selection on inbreeding.

## Methods

### Torbiscal line pedigree

The pigs of this study belong to a composite line (Torbiscal) resulting from the blending of four ancient Spanish and Portuguese strains of Iberian breeds in 1944 that was genetically isolated from 1963 to 2013 [20]. A complete genealogy of 4524 entries (individual-sire-dam) was available. The inbreeding coefficient (*F*_*ped*_) and the number of discrete generation equivalents (*EqG*) [21] were calculated based on this pedigree for each individual with respect to a base generation of unknown parents [22]. A partition of individual inbreeding coefficients into two components, new (*F*_*ped*-*new*_) and old (*F*_*ped*-*old*_), was performed using the expressions proposed by Hinrichs et al. [23]:

$$\begin{aligned} F_{i,old} \left( {u,t} \right) = \left[ {F_{i} \left( {0,t} \right) - F_{i} \left( {u,t} \right)} \right]/\left[ {1 - F_{i} \left( {u,t} \right)} \right] \hfill \end{aligned}$$

and

$$\begin{aligned} F_{i,new} \left( {u,t} \right) = F_{i} \left( {0,t} \right) - F_{i,old} \left( {u,t} \right)\quad {\text{for}}\quad 0 < u < t, \hfill \\ \end{aligned}$$

where 0 is the base generation, *u* is any intermediate generation, and *t* is the generation of the *i*-*th* individual. The intermediate generation (*u*) constituted a base by assuming that parents of pigs born in 1980 were unknown. The comparison between different pedigree and genomic inbreeding metrics was performed on data consisting of 217 sows from three related cohorts: 54 sows born between 1994 and 1998 (C1 group) with an average number of discrete generation equivalents *EqG*_*C1*_ = 21.04 (SD = 0.57) and an average pedigree inbreeding coefficient *F*_*C1*_ = 0.15 (SD = 0.01), a group of 54 sows (S) contemporary to the C1 group coming from a sub-line experimentally selected for lean growth along seven generations with *EqG*_*S*_ = 22.72 (SD = 0.78) and *F*_*S*_ = 0.21 (SD = 0.019), and a third group of 109 sows (C2) descendent from the C1 group that were born between 2004 and 2010 and with *EqG*_*C2*_ = 26.13 (SD = 0.74) and *F*_*C2*_ = 0.18 (SD = 0.02). Details of the selection experiment based on records of backfat thickness and growth can be found in Rodriguez et al. [24].

### Genotyping and SNP-based metrics of inbreeding

DNA was isolated from blood using a standard phenol/chloroform protocol and genotyped with the Illumina Porcine SNP60 BeadChip [25] and the Infinium HD Assay Ultra protocol (Illumina Inc.). Genotypes of 62,163 SNPs were called with the GenomeStudio software (Illumina). In addition, DNA from 17 Iberian pigs representing the main breeding nuclei of this breed were analyzed to identify SNPs of good quality that were monomorphic or had very low minor allele frequency (MAF) in the Torbiscal line. Quality control of genotypes was performed according to the following criteria: call rate for the individual >0.96; SNPs with a call rate >0.99; GenTrain score (measure of the reliability of the SNP detection based on the distribution of genotypic classes) >0.70; AB R mean (mean of the normalized intensity of the heterozygote cluster) >0.35; and MAF >0.05. SNPs located on sex chromosomes, those not mapped in the Sscrofa10.2 assembly (http://gbi.agrsci.dk/pig/sscrofa10_2_annotation/), or those with inconsistent inheritance from dam to daughter were also removed. Based on these criteria 35,023 SNPs were retained and used for further analyses.

### Genomic inbreeding coefficients based on the distribution of the length of ROH

A minimum number of contiguous SNPs with homozygous genotypes are required for declaring a stretch of DNA as a ROH in an individual because short tracts of homozygosity are rather common due to strong linkage disequilibrium. ROH length can be expressed either as the number of contiguous homozygous SNPs, or as the length measured in units of physical distance in Mb. These two measures of ROH length are highly correlated and both represent estimates of autozygosity (two chromosomal segments inherited from each parent that are identical from a common ancestor) since only a limited number of SNPs are genotyped within a DNA segment. Because of the exploratory nature of this paper, several alternative minimum numbers of contiguous SNPs (5, 15, 25, and 35) were used to define a ROH in order to investigate their impact on the novel inbreeding coefficients based either on the length of ROH estimated as the number of contiguous homozygous SNPs or in physical distance (Mb). For the majority of the methods, estimates of individual autozygosity (I-ROH) were taken as a deviation from a reference population or group (A-ROH). Unless stated otherwise, the reference population will consist of all individuals with available genotypes. Source code in R language (http://www.r-project.org/) for estimating the inbreeding coefficients and a small example for two individuals are provided as supplementary material in Additional files 1, 2, 3, 4  and 5.

### KS-ROH inbreeding coefficient

The Kolmogorov–Smirnov test (KS test) is a non-parametric test to compare two one-dimensional probability distributions. The Kolmogorov–Smirnov statistic (*D*) quantifies the distance between a given cumulative distribution (T) and the cumulative distribution of a reference distribution (S) and is computed as:

$$D = max_{x} \left| {F_{T} (x) - F_{S} (x)} \right|,$$

where *F*_*T*_ (*x*) and *F*_*S*_ (*x*) are the empirical cumulative distributions of T and S at point *x*, respectively. Therefore, *D* measures the largest distance between the two cumulative distribution functions. We used a modification of the KS test to compute the inbreeding coefficient of an individual based on the length of ROH by computing the KS statistic of the distribution of the lengths of ROH of the individual compared to a reference distribution that consists of the lengths of all ROH of all individuals (e.g., population, strain). Thus, the KS inbreeding coefficient measures the maximum distance between two cumulative distribution functions (CDF):

$$F_{ROH - KS} = max_{x} (F_{I - ROH} (x) - F_{A - ROH} (x)),$$

where $F_{I - ROH} (x)$ and $F_{A - ROH} (x)$ are the cumulative distributions at point x that has the maximum distance between the two distributions. The subscripts I-ROH and A-ROH refer to individual and all individuals (or reference population), respectively. Note that the absolute value was dropped from this test to allow for positive or negative deviations from the reference population.

### Quantile-ROH inbreeding coefficient

Quantiles are points taken at regular intervals from the CDF of a random variable. The *ROH*-*Q* inbreeding coefficient of an individual is defined as the sum of the differences between the quantiles of the CDF of ROH lengths of the individual (*q*_*I*-*ROH*_) and the quantiles of the reference population (*q*_*A*-*ROH*_):

$$F_{ROH - Q} = \mathop \sum \limits_{i = 1}^{nq} \frac{{q_{I - ROH} - q_{A - ROH} }}{\sqrt 2 },$$

where *nq* is the number of quantiles, *q*_*I*-*ROH*_ and *q*_*A*-*ROH*_ are the quantiles of the CDF of ROH length of the individual and of a reference population. This equation can be obtained by considering points in a Q–Q plot with coordinates *q*_*I*-*ROH*_ and *q*_*A*-*ROH*_, and measuring their distance to the diagonal (which is the expected distribution if both CDF would be equal; i.e. *f* (*x*) = *x*). The sum of all distances is *F*_*ROH*-*Q*_. Note that the distance between each point coordinate and the diagonal line (*f*(*x*) = *x*) can be positive or negative. Percentiles were the quantiles used in this study.

### Exponential-ROH inbreeding coefficient

Following Clark [26], the distribution of the length of an autozygous segment is expected to follow an exponential distribution. After fitting an exponential distribution to the distribution of the ROH lengths of an individual and of all individuals (reference population), the coefficient of inbreeding of the individual can be estimated based on the following statistics:

- Using the mean of the exponential density:
  $$F_{{ROH - E^{m} }} = \frac{1}{{\lambda_{I - ROH} }} - \frac{1}{{\lambda_{A - ROH} }},$$
  where $\lambda_{I - ROH}$ and $\lambda_{A - ROH}$ are the rates of the fitted exponential distribution for the individual and for all individuals, respectively.

(b) Using the variance of the exponential density:
  $$F_{{ROH - E^{v} }} = \frac{1}{{\lambda_{I - ROH}^{2} }} - \frac{1}{{\lambda_{A - ROH}^{2} }}.$$

(c) Using the integral of the fitted exponential density for the individual from a threshold T to ∞ to calculate the inbreeding coefficient of the individual based on the probability of getting an ROH fragment with length larger than T as:
  $$F_{{ROH - E^{p} }} = \int_{T}^{\infty } \lambda_{I - ROH} e^{{ - (\lambda_{I - ROH} )x}} d(x),$$
  where T is the threshold and x the length of the ROH. This genomic inbreeding coefficient is an estimate of the degree of autozygosity of an individual, and being a probability, it is forced to range from 0 to 1. The threshold is arbitrary but comparison between individuals is feasible when the same threshold is used for individuals from the same population typed with the same array.

Note that in the above equations, terms that apply to all individuals (i.e. the exponential distribution with rate $\lambda_{A - ROH}$) is the same for all individuals and, therefore, does not affect the ranking of individuals based on their inbreeding coefficients. Results will be provided for all three coefficients comparing correlations of these coefficients with traditional inbreeding coefficients. However, only $F_{{ROH - E^{m} }}$ or $F_{{ROH - E^{p} }}$ coefficients will be discussed in other sections of the paper in order to reduce the number of tables and figures.

### Traditional inbreeding coefficients

Correlations were estimated between the new inbreeding coefficients based on the length of ROH and the following traditional inbreeding coefficients: (1) pedigree-based inbreeding coefficient (*F*_*ped*_) computed for each individual by tracing the pedigree back to the founder animals; (2) pedigree-based new inbreeding coefficient (*F*_*ped*-new_) based on the equations proposed by Hinrichs et al. [23] with breeding animals born in 1980 as the intermediate base generation; (3) pedigree-based old inbreeding coefficient (*F*_*ped*-old_) based on the equations proposed by Hinrichs et al. [23] after ignoring all inbreeding generated from 1980 on; (4) molecular inbreeding coefficient (*F*_*Mol*_), defined as the proportion of genotyped SNPs at which an individual is homozygous (identical by state) [6]; and (5) total ROH content based metric of homozygosity [11] calculated for the autosomal genome as ${F_{ROH} = L_{ROH} /L_{AUTO} }$, where ${L_{ROH} }$ is the total ROH length of the individual and ${L_{AUTO} }$ is the length of the autosomal genome [11]. Identification of ROH was performed with the program PLINK (http://pngu.mgh.harvard.edu/purcell/plink/). In order to adapt to the much lower density of SNPs than those used by McQuillan [11], the conditions for declaring a ROH included a sliding window of 15 SNPs, allowing two missing calls and one heterozygous SNP per window; a ROH was declared if it had a length of at least 100 kb and contained 15 or more SNPs. The minimum required density was one SNP per 500 kb and the maximum gap allowed between any two consecutive SNPs was 1000 kb. Other options were according to the default settings in the program.

## Results

**Fig. 2:**
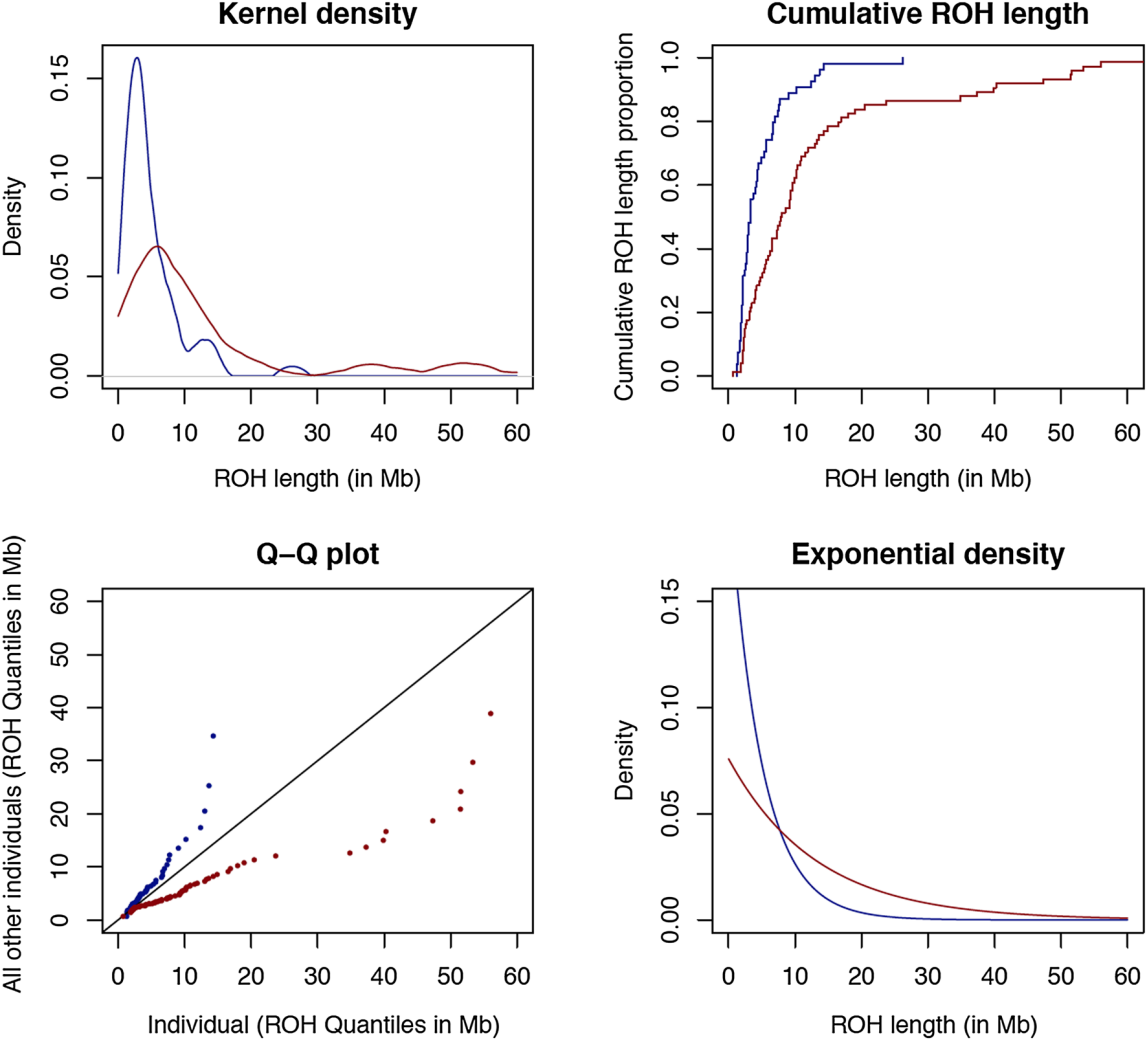
Distributional properties of the distribution of the length of runs of homozygosity (ROH). Kernel density, Q–Q plots, cumulative ROH length, and adjusted exponential density for the distributions of the length of ROH of two individuals with extreme pedigree inbreeding coefficients: sow 18705308 (highest inbreeding; *dark red*) and sow 13304804 (lowest inbreeding; *dark blue*). Minimum number of SNPs to declare a ROH >35, ROH measured in Mb

Figure 2 shows the distributional properties of two extreme sows with pedigree inbreeding coefficients of 0.13 (sow 13304804) and 0.38 (sow 18705308) to illustrate the statistical principles of the newly developed metrics. The probability densities of ROH lengths for the two sows were clearly different, with sow 18705308 having longer ROH fragments. This is also shown when plotting the cumulative distribution functions of ROH lengths for the two sows. The Kolmogorov–Smirnov method measures the maximum distance between two cumulative distributions and the observed differences in the cumulative distribution for the ROH lengths of these two individuals justify this metric. In the same manner, the two Q–Q plots for these two sows, which are graphical representations of the quantiles of each sow versus the quantiles of all sows in the dataset, show very large differences in the distributions for these two sows. The proposed quantile inbreeding coefficient is calculated by summing up the distances between the quantiles of the distribution of ROH lengths for a sow (points in the graph) and the diagonal. The shape of the line that represents the Q–Q plots, i.e. whether it is curved or linear, may exemplify episodes of recent or old inbreeding. Finally, the adjusted exponential densities of the two sows show differences in the rate at which the highly inbred sow has longer ROH fragments. The right hand side of this graph is similar to the observed kernel density, whereas the distribution to the left of the peak does not seem to fit an exponential density. In summary, there are differences in the distribution of ROH lengths between individuals depending on their inbreeding history.

**Fig. 3:**
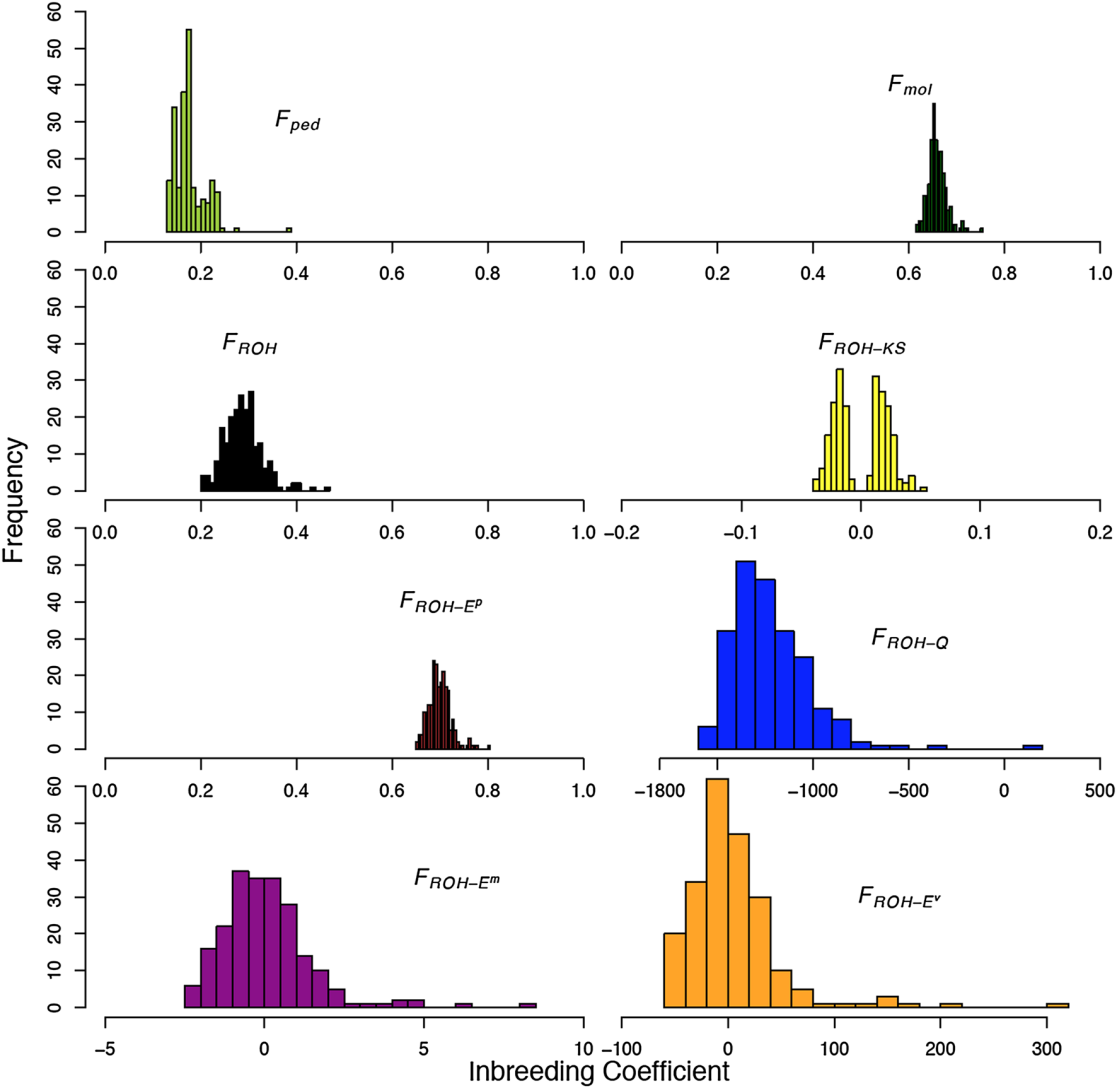
Histograms of frequencies of pedigree and genomic inbreeding coefficients. The histograms are based on ROH lengths from 217 Torbiscal Iberian sows (minimum number of SNPs to declare a ROH >5 for quantile and exponential, ROH-length in Mb). The threshold for exponential-*p* was 5 Mb. Abbreviations for inbreeding coefficients: *F*_*ped*_, pedigree; *F*_*mol*_, molecular; *F*_*ROH*_, total ROH content; *F*_*ROH*-*KS*_, Kolmolgorov–Smirnov; *F*_*ROH*-*E*_^*p*^, probability exponential; *F*_*ROH*-*Q*_, quantile; *F*_*ROH*-*E*_^*m*^, mean exponential; *F*_*ROH*-*E*_^*v*^, variance exponential

Figure 3 shows the histograms of pedigree and ROH-based inbreeding coefficients to better picture the distribution of the novel inbreeding coefficients. The distributions of both *F*_*ped*_ and *F*_*Mol*_, with respective means of 0.18 (SD = 0.03) and 0.74 (SD = 0.02), are similar but have a different range of values. All ROH-based coefficients, except *F*_*ROH*-*E*_^*p*^, have values that fall outside the range of 0–1, which is problematic when comparing inbreeding coefficients between populations or species. Both traditional and novel inbreeding coefficients were able to identify the sow with the highest inbreeding (18705308), i.e., the most extreme value on the right hand side of all histograms.

In order to illustrate the new metrics, correlations between traditional and ROH-based inbreeding coefficients are in Tables 1 and 2 when considering length of ROH either as the number of SNPs (Table 1) or as physical distance in Mb (Table 2). Correlations were computed for a range of minimum number of SNPs (5, 15, 25, and 35) in a DNA stretch tested for autozygosity. The correlation between ROH-based and pedigree (*F*_*ped*_) inbreeding coefficients ranged from 0.22 to 0.72 (Table 1) and from 0.17 to 0.71 (Table 2). All correlations were lower when ROH length was measured in Mb. Partitioning individual inbreeding coefficients into new (*F*_*ped*-*new*_) and old (*F*_*ped*-*old*_) inbreeding, allowed the proposed ROH metrics to be correlated with recent and past inbreeding. For all ROH inbreeding coefficients, regardless of the minimum number of SNPs used and whether ROH length was based on the number of SNPs or physical distance, the correlations of the novel ROH metrics with *F*_*ped*-*new*_ were very similar to their correlations with *F*_*ped*_, whereas the correlation with *F*_*ped*-*old*_ was small. Except for *F*_*ROH*-*KS*_ (minimum number of SNPs >5), the novel metrics were also highly correlated with *F*_*Mol*_ and *F*_*ROH*_, as expected. Correlations of the parametric inbreeding coefficients *F*_*ROH*-*E*_^*m*^, *F*_*ROH*-*E*_^*v*^ and *F*_*ROH*-*E*_^*p*^ with *F*_*ped*_, *F*_*Mol*_ and *F*_*ROH*_ were higher when the minimum number of SNPs to determine ROH was equal to five, which is as expected since the use of a larger number of SNPs truncates the parametric distribution. In contrast, correlations of inbreeding coefficients based on the non-parametric distributions *F*_*ROH*-*KS*_ and *F*_*ROH*-*Q*_, with *F*_*ped*_, *F*_*Mol*_ and *F*_*ROH*_ were higher for an intermediate minimum number of SNPs. For example, the KS inbreeding coefficient gave a low correlation with pedigree inbreeding coefficients of 0.22, 0.47, 0.50 and 0.47 for minimum number of SNPs equal to 5, 15, 25, and 35, respectively (Table 1). This method uses the largest distance between the two cumulative distributions, which may be better estimated by ignoring small ROH fragments. For ROH with a minimum number of SNPs greater than 5, correlations between *F*_*Mol*_ and inbreeding coefficients based on the length of ROH ranged from 0.41 to 0.97 (Table 1), and from 0.32 to 0.96 (Table 2). Similarly, total ROH content was highly correlated with all new inbreeding coefficients, with the exception of *F*_*ROH*-*KS*_ for a minimum number of SNPs greater than 5.

**Table 1:** Correlations between current and new genomic inbreeding coefficients measured in number of SNPs and using alternative minimum numbers of SNPs when declaring a ROH

	*F*_*ROH*-*KS*_	*F*_*ROH*-*Q*_	*F*_*ROH*-*E*_^*m*^	*F*_*ROH*-*E*_^*v*^	*F*_*ROH*-*E*_^*p*^
Minimum number of SNPs >5					
*F*_*ped*_	0.223	0.548	0.716	0.723	0.690
*F*_*ped*-*new*_	0.226	0.548	0.717	0.724	0.691
*F*_*ped*-*old*_	−0.056	0.170	0.171	0.164	0.174
*F*_*Mol*_	0.406	0.749	0.963	0.945	0.969
*F*_*ROH*_	0.407	0.749	0.948	0.927	0.960
Minimum number of SNPs >15					
*F*_*ped*_	0.472	0.663	0.672	0.677	0.630
*F*_*ped*-*new*_	0.473	0.663	0.672	0.677	0.630
*F*_*ped*-*old*_	0.081	0.190	0.187	0.183	0.176
*F*_*Mol*_	0.675	0.892	0.902	0.885	0.892
*F*_*ROH*_	0.897	0.877	0.884	0.870	0.877
Minimum number of SNPs >25					
*F*_*ped*_	0.500	0.655	0.651	0.667	0.587
*F*_*ped*-*new*_	0.501	0.655	0.651	0.668	0.587
*F*_*ped*-*old*_	0.113	0.199	0.199	0.188	0.199
*F*_*Mol*_	0.688	0.866	0.857	0.850	0.830
*F*_*ROH*_	0.676	0.837	0.828	0.818	0.802
Minimum number of SNPs >35					
*F*_*ped*_	0.470	0.631	0.621	0.650	0.544
*F*_*ped*-*new*_	0.470	0.630	0.620	0.650	0.543
*F*_*ped*-*old*_	0.167	0.224	0.225	0.212	0.231
*F*_*Mol*_	0.655	0.829	0.813	0.815	0.774
*F*_*ROH*_	0.638	0.796	0.780	0.780	0.744

Current inbreeding coefficients are pedigree (*F*_*ped*_, *F*_*ped*-*new*_ and *F*_*ped*-*old*_), molecular (*F*_*Mol*_) and total ROH content ROH (*F*_*ROH*_); novel genomic inbreeding coefficients are Kolmolgorov–Smirnov (*F*_*ROH*-*KS*_), quantile (*F*_*ROH*-*Q*_), exponential mean, variance and probability (*F*_*ROH*-*E*_^*m*^, *F*_*ROH*-*E*_^*v*^, *F*_*ROH*-*E*_^*p*^); standard errors range from 0.017 to 0.068

**Table 2:** Correlations between current and new genomic inbreeding coefficients measured in length of ROH in Mb and using alternative minimum numbers of SNPs when declaring a ROH

	*F*_*ROH*-*KS*_	*F*_*ROH*-*Q*_	*F*_*ROH*-*E*_^*m*^	*F*_*ROH*-*E*_^*v*^	*F*_*ROH*-*E*_^*p*^
Minimum number of SNPs >5					
*F*_*ped*_	0.166	0.510	0.701	0.713	0.704
*F*_*ped*-*new*_	0.169	0.510	0.707	0.714	0.705
*F*_*ped*-*old*_	−0.081	0.150	0.158	0.151	0.161
*F*_*Mol*_	0.317	0.696	0.960	0.939	0.960
*F*_*ROH*_	0.295	0.717	0.956	0.931	0.956
Minimum number of SNPs >15					
*F*_*ped*_	0.447	0.645	0.652	0.656	0.625
*F*_*ped*-*new*_	0.447	0.645	0.653	0.657	0.625
*F*_*ped*-*old*_	0.110	0.176	0.176	0.171	0.172
*F*_*Mol*_	0.613	0.878	0.894	0.875	0.890
*F*_*ROH*_	0.897	0.883	0.893	0.865	0.893
Minimum number of SNPs >25					
*F*_*ped*_	0.459	0.618	0.614	0.631	0.561
*F*_*ped*-*new*_	0.458	0.619	0.614	0.632	0.560
*F*_*ped*-*old*_	0.168	0.184	0.185	0.171	0.194
*F*_*Mol*_	0.587	0.850	0.840	0.835	0.813
*F*_*ROH*_	0.580	0.843	0.832	0.823	0.808
Minimum number of SNPs >35					
*F*_*ped*_	0.445	0.586	0.576	0.607	0.507
*F*_*ped*-*new*_	0.443	0.585	0.576	0.607	0.506
*F*_*ped*-*old*_	0.244	0.197	0.198	0.182	0.140
*F*_*Mol*_	0.607	0.809	0.792	0.797	0.675
*F*_*ROH*_	0.599	0.801	0.794	0.786	0.747

Current inbreeding coefficients are pedigree (*F*_*ped*_, *F*_*ped*-*new*_ and *F*_*ped*-*old*_), molecular (*F*_*Mol*_) and total ROH content (*F*_*ROH*_); novel genomic inbreeding coefficients are Kolmolgorov–Smirnov (*F*_*ROH*-*KS*_), quantile (*F*_*ROH*-*Q*_ *)* and exponential mean, variance and probability (*F*_*ROH*-*E*_^*m*^, *F*_*ROH*-*E*_^*v*^, *F*_*ROH*-*E*_^*p*^); standard errors range from 0.015 to 0.068

**Fig. 4:**
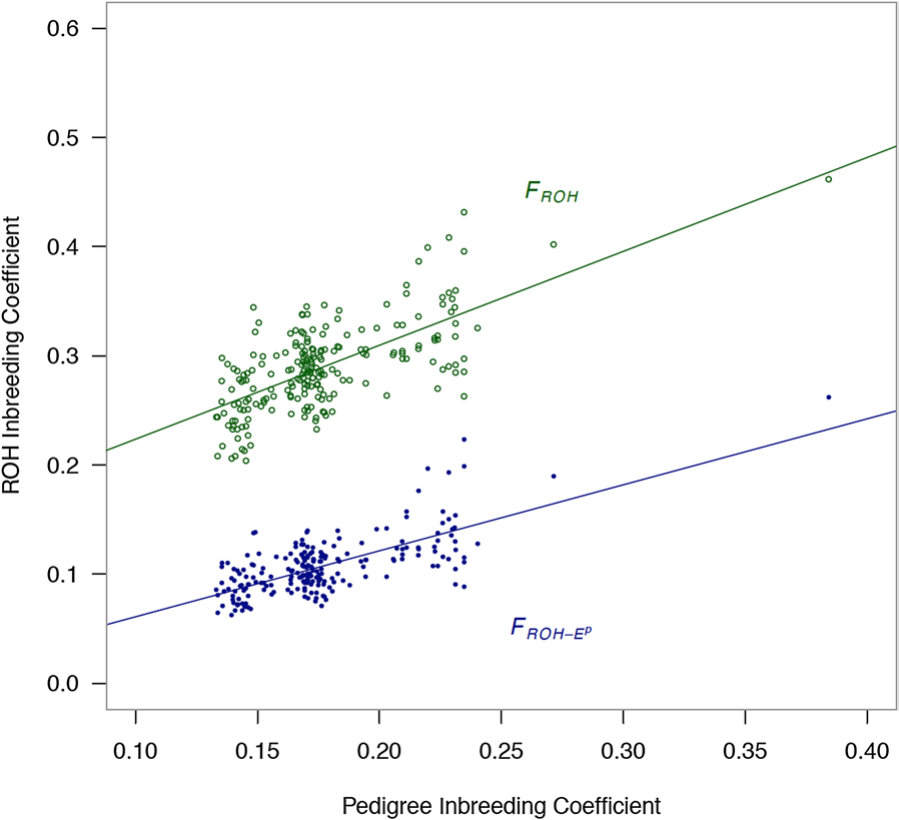
Regressions of *F*_*ROH*-*E*_^*p*^ and *F*_*ROH*_ on pedigree inbreeding coefficients (*F*_*ped*_). Minimum number of SNPs to declare a ROH >5 for *F*_*ROH*-*E*_^*p*^ and T = 2 Mb and ROH measured in Mb

**Fig. 5:**
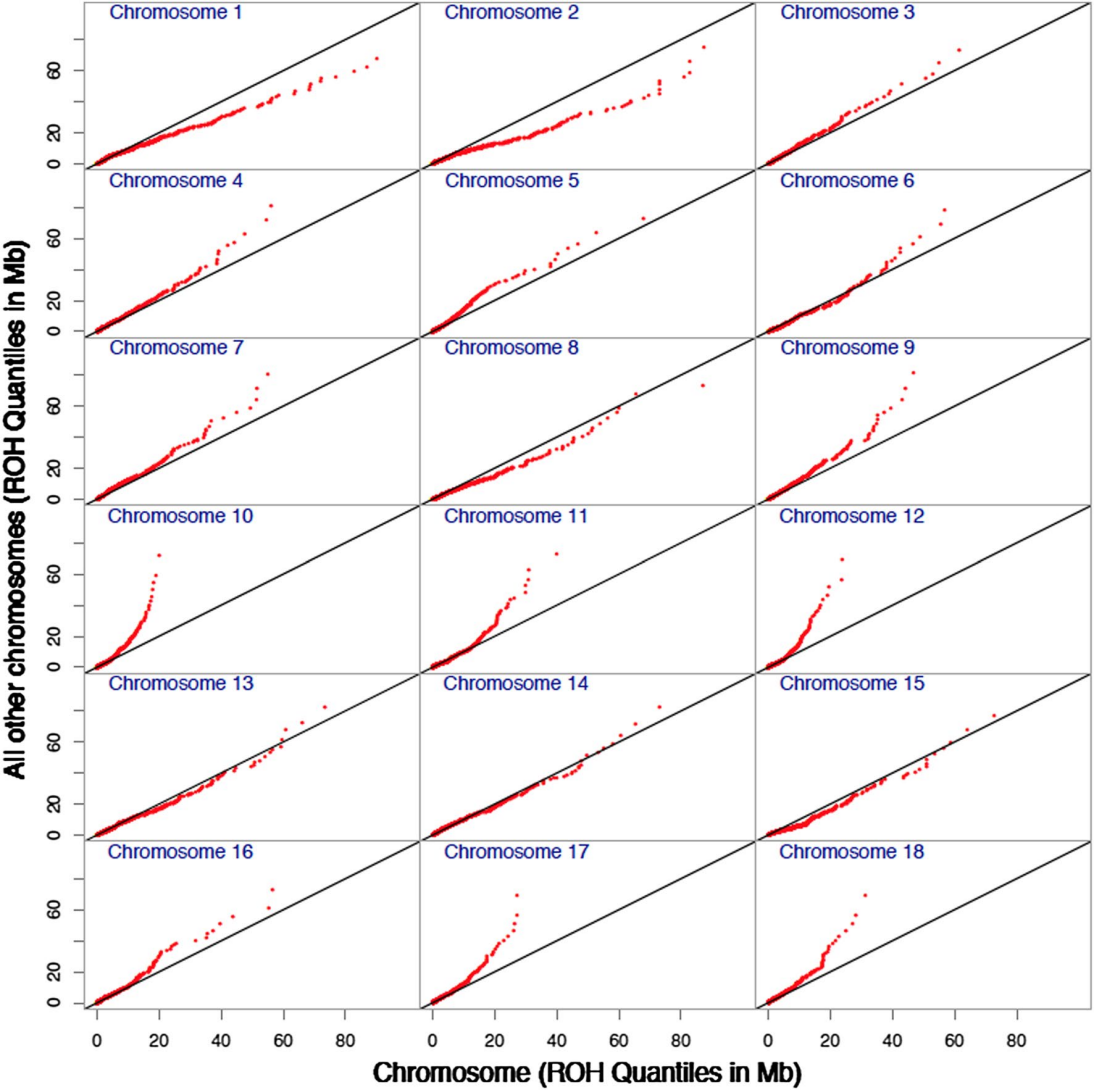
Q–Q plots of the distribution of the length of ROH for each of the 18 autosomes versus all chromosomes. The chromosome being tested was not included in the reference population; minimum number of SNPs to declare a ROH >5, ROH measured in Mb

Figure 4 shows the regression of pedigree-based inbreeding coefficients on *F*_*ROH*-*E*_^*p*^ (correlation = 0.70) and on total ROH content inbreeding coefficients (correlation = 0.68). Some values of *F*_*ped*_ correspond to many values for *F*_*ROH*-*E*_^*p*^ and *F*_*ROH*_, suggesting that ROH inbreeding coefficients incorporate segregation within families, in contrast to the pedigree-based coefficients. The three groups of sows (S, C1, and C2) can also be distinguished as clusters of dots in each of the two regressions. The use of the distribution of ROH-length is not restricted to the estimation of the individual inbreeding coefficients but it can also be used to explore differences in inbreeding on different chromosomes. Table 3 provides correlations between chromosomal length and genomic inbreeding coefficients, which show that chromosome length is correlated with *F*_*ROH*-*Q*_ and to a lesser extent with *F*_*ROH*-*E*_^*p*^ and *F*_*ROH*-*E*_^*m*^, suggesting that these inbreeding coefficients are very much affected by chromosomal length. A very high correlation between *F*_*ROH*-*E*_^*p*^ and *F*_*ROH*-*E*_^*m*^ was observed, which may be due to the high dependency of these two parameters on the “rate” of the exponential distribution. Figure 5 shows Q–Q plots of the distribution of quantiles for each chromosome when plotted against the distribution for all other chromosomes from all individuals. Chromosome 1 had the greatest *F*_*ROH*-*Q*_ values, while chromosome 10 had the lowest *F*_*ROH*-*Q*_ values and ROH fragments that were shorter than those for the rest of the genome. These results illustrate the relationship between *F*_*ROH*-*Q*_ and chromosomal length.

**Table 3:** Correlations between pairs of chromosomal genomic inbreeding coefficients and length (number of SNPs per chromosome)

	Length	*F*_*Mol*_	*F*_*ROH*_	*F*_*ROH*-*KS*_	*F*_*ROH*-*Q*_	*F*_*ROH*-*E*_^*m*^	*F*_*ROH*-*E*_^*p*^
Length		0.04	0.16	0.57	0.84	0.57	0.57
*F*_*Mol*_	0.25		0.45	0.01	−0.07	0.44	0.45
*F*_*ROH*_	0.25	0.25		0.32	0.13	0.51	0.52
*F*_*ROH*-*KS*_	0.21	0.25	0.24		0.60	0.83	0.82
*F*_*ROH*-*Q*_	0.14	0.25	0.25	0.20		0.59	0.58
*F*_*ROH*-*E*_^*m*^	0.20	0.22	0.21	0.14	0.20		0.99
*F*_*ROH*-*E*_^*p*^	0.21	0.22	0.21	0.14	0.20	0.00	

Correlations are on the upper diagonal; approximate standard errors are on the lower diagonal; *F*_*ROH*-*KS*_ *, F*_*ROH*-*Q*_, *F*_*ROH*-*E*_^*m*^, and *F*_*ROH*-*E*_^*p*^ were computed for a minimum of five SNPs and measured as physical distance in Mb; quantiles in *F*_*ROH*-*Q*_ were computed excluding the chromosome being tested from the reference population; *F*_*ROH*-*E*_^*p*^ were computed for a threshold of T = 5 Mb

**Fig. 6:**
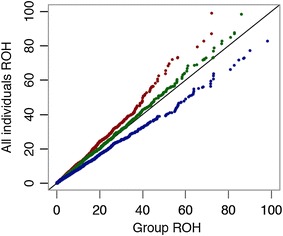
The effect of selection on the distribution of the length of ROH. Q–Q plots of the distribution of ROH fragment sizes for all individuals versus the distribution for individuals C1 (*dark-red*), C2 (*dark-green*), and S (*dark-blue*); minimum number of SNPs to declare a ROH >5, ROH measured in Mb

The use of the distribution of ROH length to measure inbreeding does not only open new possibilities to investigate autozygosity at the chromosomal level but also to investigate old established principles and knowledge on inbreeding. In 1961, Alan Robertson [27] postulated that inbreeding must increase in populations under selection. Figure 6 shows Q–Q plots of the distribution of the length of ROH for the unselected groups C1 and C2 and for the selected group S relative to the ROH distribution of all individuals. A clear pattern can be observed, showing that the S group had systematically larger ROH fragments, and therefore, more inbreeding than groups C1 and C2. Group C2 had larger ROH fragments than C1, which is attributable to an increase in inbreeding during the five generations that passed between C1 sows and their descendants in C2. There was also an increase in pedigree inbreeding coefficients in the selected group S (0.21) relative to C1 (0.14) and C2 (0.17). This increase in inbreeding was also evident for *F*_*ROH*_ (0.21 for C1, 0.25 for C2, and 0.28 for S) or for *F*_*Mol*_ (0.65 for C1, 0.65 for C2, and 0.67 for S). Similarly, exponential inbreeding coefficients were higher for the selected group, S. In summary, selection has impacted the genetic variability of the S group, as detected by both pedigree and molecular-based indicators of inbreeding.

**Fig. 7:**
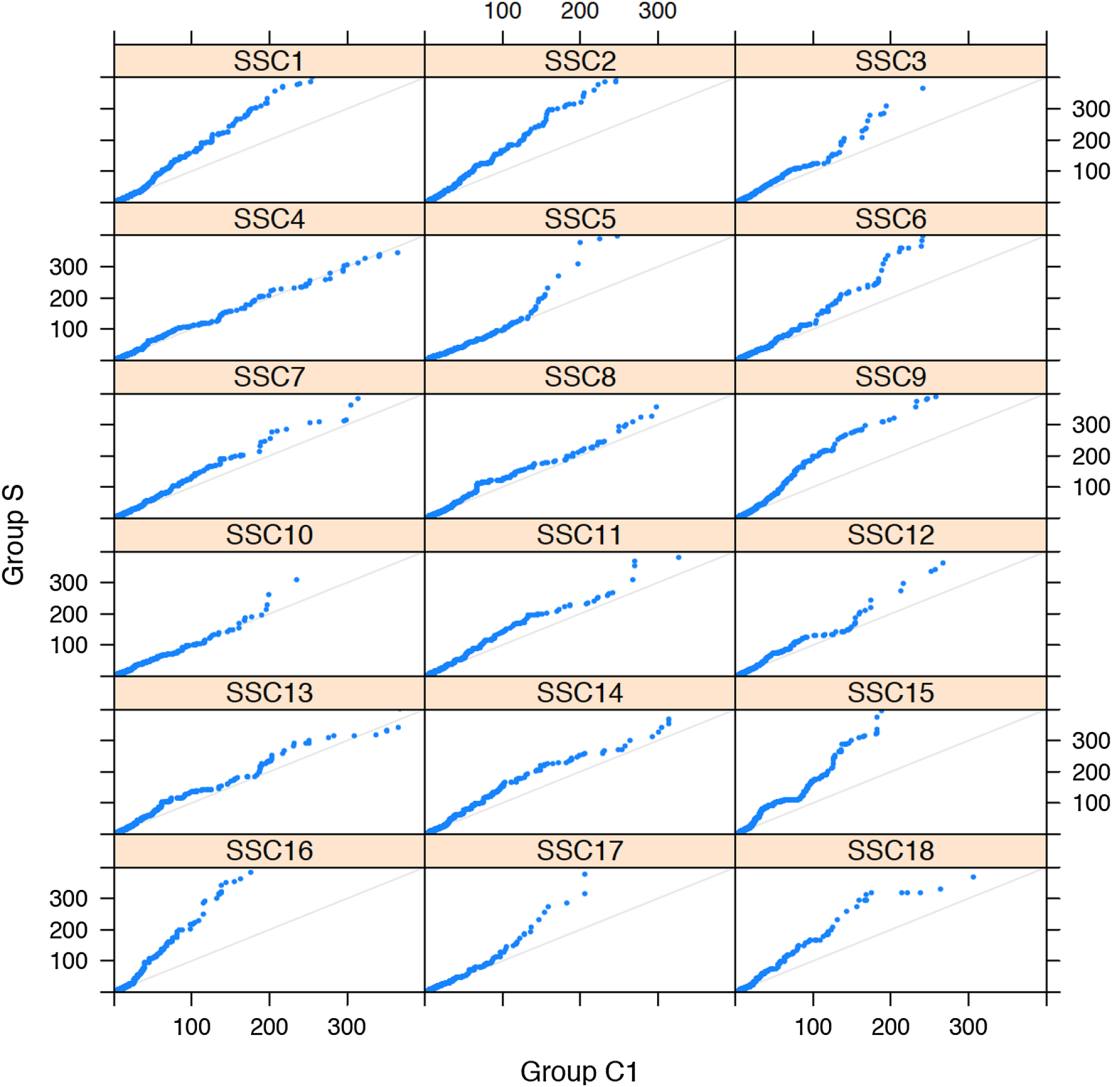
Q–Q plots of the distribution of ROH for selected group S versus group C1 for each of the 18 autosomes. Minimum number of SNPs to declare a ROH >5, ROH-length measured by the number of contiguous homozygous SNPs

**Fig. 8:**
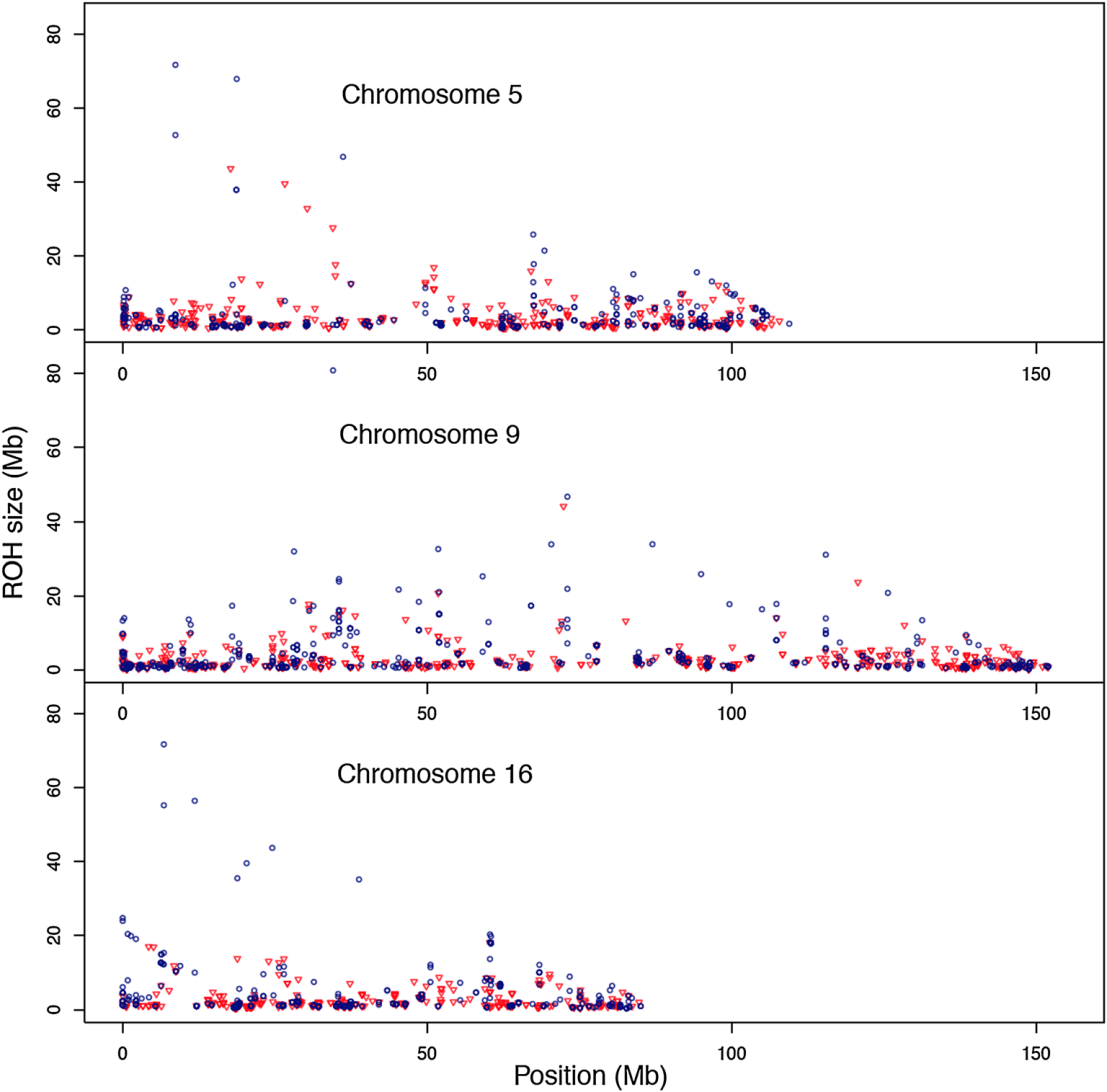
Distribution of ROH-length along chromosomal positions. The *figure* represents conservation group (C1 *color red*), and selected group (S *color navy*) on chromosomes 5, 9, and 16 for a minimum number of SNPs to declare a ROH >15 and measured in Mb

The next question was to investigate whether the increase in length of ROH was more marked in some chromosomes as a result of selection. Figure 7 shows Q–Q plots of the S group versus the non-selected C1 group by chromosome. Most chromosomes had longer ROH in the S group. The distributions in both groups were similar for chromosome 4, while the C1 group had some larger ROH fragments for chromosome 13. Figure 8 shows the length of each ROH along their position on chromosomes 5, 9 and 16. These chromosomes were chosen because of the larger sizes of ROH fragments in the S group versus C1 (Fig. 7). There were ROH of larger sizes at the beginning of chromosomes 5 and 16. Large ROH fragments were distributed evenly along chromosome 9.

## Discussion

By “genomic inbreeding coefficient”, we denote a parameter that uses genomic information on autozygosity as a measure of relatedness among ancestors of an individual. It includes molecular inbreeding coefficients, ROH inbreeding coefficients [10–19, 28, 29] and coefficients that make use of the length of ROH as a random variable with an associated probability distribution or probability density function, as proposed in this paper. One of the first issues that had to be addressed is how to estimate ROH. DNA sequencing methods are required to observe autozygosity but often ROH are estimated based on genotypes obtained with BeadChip arrays of SNPs. Stretches of DNA are declared as ROH if a minimum number of consecutive SNPs from an array are homozygous. We explored four different minimum numbers of SNPs to declare a ROH (5, 15, 25, and 35) and considered two alternative measures of length, the number of SNPs and physical length in Mb. Our results suggest that the minimum number of SNPs can affect correlations between genomic and pedigree inbreeding coefficients. On the one hand, Quantile and Kolmolgorov–Smirnov ROH inbreeding coefficients were less correlated with pedigree inbreeding coefficients when the minimum number was small, in contrast to exponential inbreeding coefficients. Nevertheless, differences between inbreeding coefficients based on ROH length were not large, except for *F*_*ROH*-*KS*_. On the other hand, correlations between pedigree and genomic inbreeding coefficients were slightly higher when using the ROH length based on number of SNPs instead of physical distance. An explanation is that only some SNPs in a DNA fragment are genotyped and errors in declaring a fragment autozygous add another source of error to the usual genotyping errors, such as SNP location or distance between SNPs in the array. Nevertheless, the correlations based either on the number of SNPs or on physical distance were rather similar across all situations investigated.

All inbreeding coefficients (traditional and newly developed) have advantages and disadvantages. The advantage of the pedigree inbreeding coefficient is that it is simple and only requires recording of pedigrees but it does not account for the sampling that occurs when gametes are produced during meiosis. That is, pedigree inbreeding coefficients are probabilistic and do not account for the fact that individuals with the same inbreeding history can differ in autozygosity. For example, two full-sibs can have different numbers of fragments of autozygosity (and at different locations) just by sampling. In contrast, all genomic inbreeding coefficients account for sampling and they measure the “realized inbreeding” of an individual.

Genomic inbreeding coefficients differ in the way they use the genotype information. Molecular inbreeding coefficients are calculated as the proportion of homozygous sites that are genotyped with an array. They assume that the genotyped SNPs are randomly located across the genome and do not distinguish old from recent inbreeding. This coefficient incorporates the entire breeding history of the individual, including new mutations and old inbreeding. The total ROH content inbreeding coefficient is the proportion of the genome of an individual that comprises autozygous fragments. This coefficient does incorporate regions of autozygosity but, in contrast to the molecular coefficient, it ignores fragments consisting of a single or a few contiguous homozygous SNPs in its computation. Total ROH content inbreeding coefficient does distinguish old from recent inbreeding but with the limitation that direct information on the length of ROH fragments is not used. In principle, two individuals with the same total ROH content inbreeding coefficients can have a different proportion of large and short ROH fragments. However, total ROH content inbreeding coefficients may indirectly account for the length of ROH because highly inbred animals, such as progeny from the mating between two full-sibs, should have larger total ROH content inbreeding coefficients made up by a large number of ROH of larger size.

The inbreeding coefficients proposed in this paper do incorporate direct information on the length of ROH to a greater or lesser extent. The Kolmogorov–Smirnov coefficient is a general method to compare statistical distributions and was used here to discern individuals with a very different distribution of ROH length when compared to the rest of the population. The Quantile inbreeding coefficient is very well suited to detect individuals with larger ROH fragments due to recent inbreeding and it leads to a graphical representation of the inbreeding of an individual. The exponential mean and variance inbreeding coefficients assume that one single parameter, the rate of the exponential distribution, defines the inbreeding status of an individual. A higher rate means that the individual has a greater average length of ROH fragments. For simplicity, the reference population was a pool of all ROH fragments of all individuals. This part corresponding to the reference population could be better represented by fitting a gamma distribution instead of an exponential distribution since the sum of exponentials follows this distribution. Kolmogorov–Smirnov, Quantile, and exponential inbreeding coefficient do not fall within the range of 0–1 (in contrast to the pedigree, molecular, and total ROH content inbreeding coefficients) but they could easily be standardized (i.e., forced to be between 0 and 1) by:

$$Standardized \,\, inbreeding \,\,of \,\,the \,\, ith \,\, individual = \frac{{F_{i}^{{}} - min(F_{ROH} )}}{{max(F_{ROH}^{{}} ) - min(F_{ROH} )}},$$

where $F_{i}^{{}}$ is the inbreeding coefficient of the *i*-th individual before it is standardized and $F_{ROH}^{{}}$ is its distribution (K–S, quantile or exponential). However, the standardized inbreeding coefficients do not abide by the definition of an inbreeding coefficient (i.e., the probability that two alleles at a locus in an individual are identical by descent), and cannot be used to compare individuals from populations with a different inbreeding history. The exponential-*p* inbreeding coefficient does range from 0 to 1, since it is defined as a probability. The exponential-*p* inbreeding coefficient requires definition of a threshold, T, which should be the same when comparing inbreeding of different individuals from the same population. More work is needed to explore the impact of alternative thresholds on estimates of inbreeding coefficients of animals from different populations.

Comparison of the new metrics to existing methods provides little information on their ability to detect long ROH (as an aid to detect recent inbreeding) since existing methods cannot. Thus, in order to investigate the ability of the new methods to detect long ROH fragments, correlations between chromosomal inbreeding and chromosomal length were performed. A recent common ancestor of the parents of an individual is expected to result in entire chromosomes or long DNA fragments (as a result of single or multiple recombination events in the different paths leading to the parents of the individual) to be identical by descent in the individual. Therefore, long chromosomes are expected to result in longer ROH fragments. In addition, longer chromosomes have been shown to have a lower recombination rate (cM/Mb) in swine [30], which would also result in longer ROH fragments. Our results show that chromosomal length was highly correlated with quantile chromosomal inbreeding coefficients and to a lesser extent with other proposed metrics. Thus, quantile inbreeding coefficients are sensitive to long ROH fragments and, therefore, improve detection of recent inbreeding.

The largest limitation of the newly proposed metrics is that they do not allow for straightforward comparison of the level of inbreeding of individuals from different species. Genomes with different number and size of chromosomes (or recombination rate) may lead to distributions of individual inbreeding coefficients based on ROH length that are not comparable. This may be overcome by using exponential-*p* inbreeding coefficients and by setting appropriate thresholds that facilitate comparisons across species. For example, thresholds could be chosen based on the distributions of ROH length for each species relative to the distribution of ROH length of several species together.

Traditional and new inbreeding coefficients allowed for the detection of the effect of selection on inbreeding [27]. However, the genomic inbreeding coefficients can pinpoint chromosomal regions where autozygosity is more extensive. Selection has two effects on inbreeding: one is its direct action to increase the frequency of alleles that favorably affect the trait under selection; the other is the increase in inbreeding and autozygosity for all loci regardless of their effects on the trait, which is attributed to co-selection of individuals with high breeding values which tend to share not just alleles at loci with an effect on the trait but at all loci, i.e., to be relatives [31]. Our results for chromosomes 5, 9 and 16 support the hypothesis that autozygosity affects both loci that are related to the selected traits as well as neutral loci scattered over the genome. In addition, the increased autozygosity in the S group is apparent for all chromosomes except chromosome 4. The method can identify chromosomal inbreeding but not the reasons for its occurrence.

In conservation genetics, coancestry coefficients are used to optimize genetic management in a conservation program and several estimators of coancestries based on molecular information have been proposed, e.g., [32, 33]. These methods ignore that linked SNPs are inherited together, and consequently, the information provided by ROH. Pryce et al. [34] showed that ROH could provide additional information on coancestry when mating relatives. However, their approach consisted in estimating the proportion of haplotypes at a given length of ROH that are common between individuals. A novel alternative would be to make use of the expected distribution of the length of the ROH among progeny of related parents, in line with our proposed use of ROH to quantify inbreeding. In other words, to use coancestry coefficients based on the expected shape of the distribution of ROH lengths in the progeny of the two parents.

## Conclusions

The proposed inbreeding coefficients add to existing methods to estimate inbreeding by accounting for the length of ROH, which incorporates information on recent inbreeding. Among the proposed metrics, quantile inbreeding coefficients are the most sensitive for identifying individuals with longer ROH fragments. Exponential-*p* inbreeding coefficients are less sensitive for detecting long ROH fragments but are defined as a probability (they range from 0 to 1) and are, therefore, suitable for comparison of individuals across populations.

## Additional files

10.1186/s12711-015-0153-1 Supplemental.Running.example.June2015.docx. This file contains instructions for running an example for the new inbreeding coefficients.
10.1186/s12711-015-0153-1 Inbreeding.example. This file contains a small dataset with information on ROH length for two individuals on chromosome 1. The variables are: nchro: number of chromosome; ind: number of individuals; gr: group; po1: initial chromosomal position; po2: final chromosomal position; roh: ROH length in number of homozygous SNPs in the segment.
10.1186/s12711-015-0153-1 k-s.r. Source code in R for estimating Kolmogorov-Smirnov inbreeding coefficients.
10.1186/s12711-015-0153-1 Quantile.r. Source code in R for estimating quantile inbreeding coefficients.
10.1186/s12711-015-0153-1 Expon.r. Source code in R for estimating exponential inbreeding coefficients.
