# Supplementary material for: Genomic inbreeding coefficients based on the distribution of the length of runs of homozygosity in a closed line of Iberian pigs
Source: Genet Sel Evol. 2015 Oct 16;47:81. doi: 10.1186/s12711-015-0153-1 (PMC4608316; doi:10.1186/s12711-015-0153-1)
Supplement: Supplementary file 1 — 10.1186/s12711-015-0153-1 Supplemental.Running.example.June2015.docx. This file contains instructions for running an example for the new inbreeding coefficients. [file 12711_2015_153_MOESM1_ESM.docx]

Supplemental Material

***Example for estimating inbreeding coefficients for two individuals.***

The file inbreeding.example contains information on the number of SNP in each ROH (roh), initial and final position (po1 and po2), chromosome number (nchro), group (gr) for two individuals (ind). The R source code for estimating inbreeding confidents is in file K-S.r (Kolmolgorov-Smirnof ROH), expon.r (exponential ROH) and quantile.r (Quantile ROH). The ROH are for SSC1 of the two individuals).

***Kolmogorov-Smirnov Inbreeding Coefficient***

To run R code just type:

- source("k-s.r")

individual 1 D^-

0.04554455

individual 2 D^+

-0.03198354

which are the inbreeding coefficients for individuals 1 and 2 (5844 and 5845) in the file inbreeding.example.

***Quantile Inbreeding Coefficient***

To run R code just type:

- source("quantile.r")

individual 1[1] 17.70897

individual 2[1] -26.32545

***Exponential Inbreeding Coefficient***

- source("expon.r")

[1] "Exponential-mean "

individual 1

Inbreeding

0.3613788

individual 2

Inbreeding

-0.2537772

[1] "Exponential-p"

individual 1

Inbreeding

0.2280106

individual 2

Inbreeding

0.06645968

There is an output file with a q-q plot in the same directory where the program is run with names K-s.pdf, quantile.pdf, and expon.pdf.


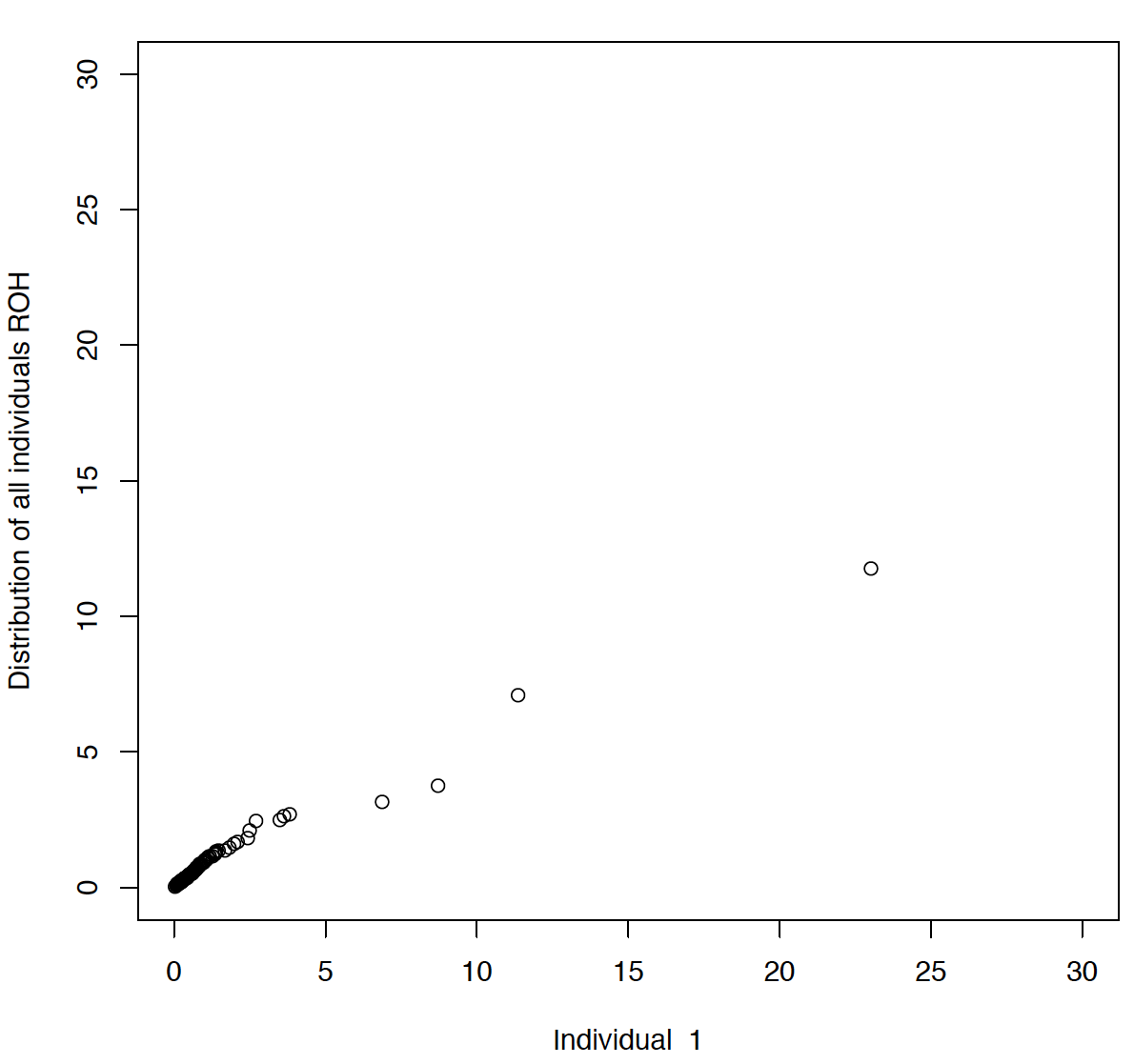


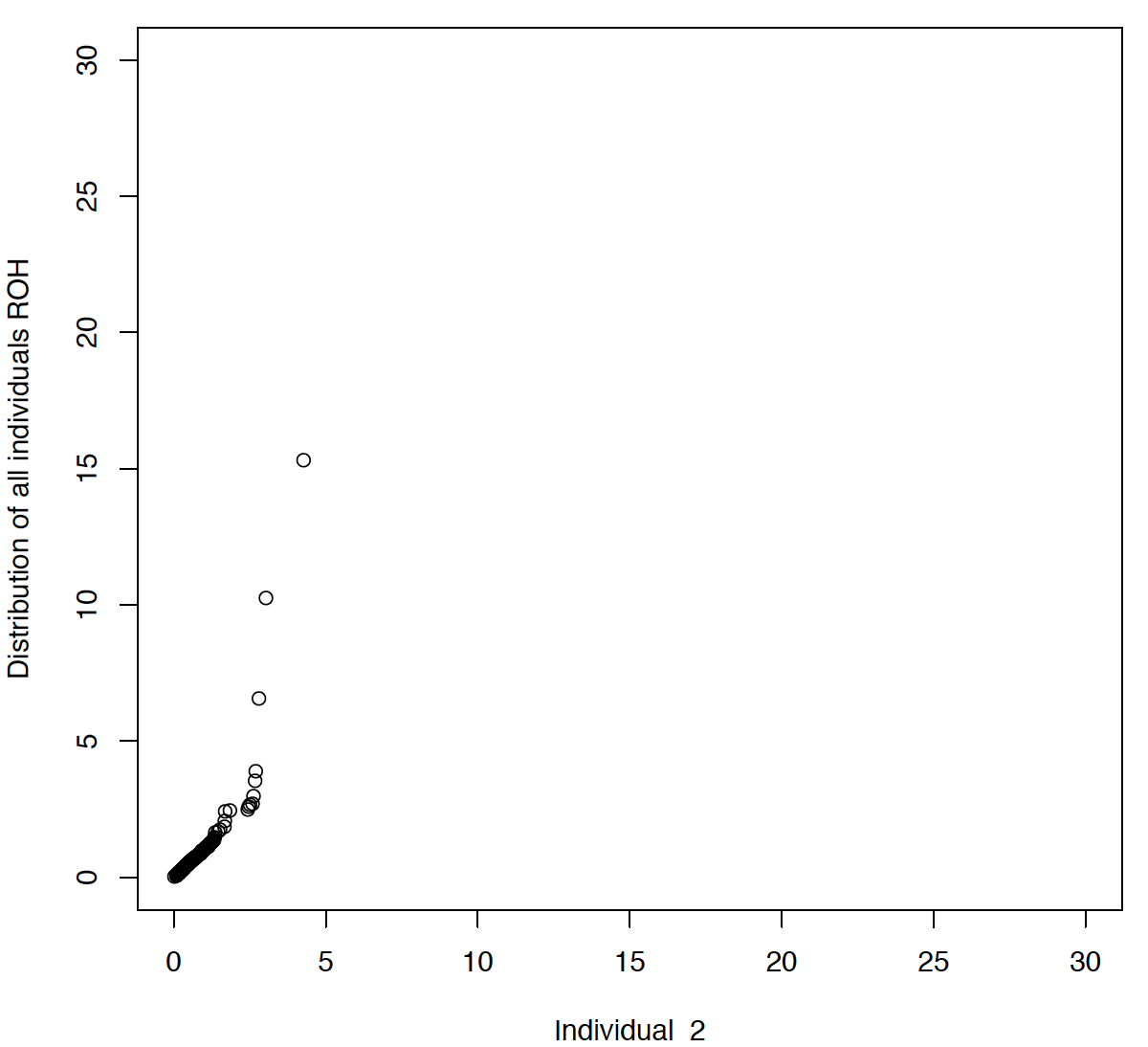


Regardless of the estimated inbreeding coefficient individual 1 has larger inbreeding coefficient than individual 2. There are options (requiring small modifications in the source code) to suppress warnings in R or to use the number of SNPs instead of length in Mb.
